# Bone Marrow-Derived Stem Cell Factor Regulates Prostate Cancer-Induced Shifts in Pre-Metastatic Niche Composition

**DOI:** 10.3389/fonc.2022.855188

**Published:** 2022-04-19

**Authors:** Brittni M. Foster, Lihong Shi, Koran S. Harris, Chirayu Patel, Victoria E. Surratt, Kendall L. Langsten, Bethany A. Kerr

**Affiliations:** ^1^ Department of Cancer Biology, Wake Forest School of Medicine, Winston-Salem, NC, United States; ^2^ Wake Forest Baptist Comprehensive Cancer Center, Winston-Salem, NC, United States

**Keywords:** stem cell factor, CD117/c-kit, prostate cancer, bone microenvironment, platelet, osteoblast

## Abstract

Skeletal metastasis is the leading cause of morbidity and mortality in prostate cancer, with 80% of advanced prostate cancer patients developing bone metastases. Before metastasis, bone remodeling occurs, stimulating pre-metastatic niche formation and bone turnover, and platelets govern this process. Stem cell factor (SCF, Kit Ligand) is increased in advanced prostate cancer patient platelet releasates. Further, SCF and its receptor, CD117/c-kit, correlate with metastatic prostate cancer severity. We hypothesized that bone-derived SCF plays an important role in prostate cancer tumor communication with the bone inducing pre-metastatic niche formation. We generated two cell-specific SCF knockout mouse models deleting SCF in either mature osteoblasts or megakaryocytes and platelets. Using two syngeneic androgen-insensitive murine prostate cancer cell lines, RM1 (*Ras* and *Myc* co-activation) and mPC3 (*Pten* and *Trp53* deletion), we examined the role of bone marrow-derived SCF in primary tumor growth and bone microenvironment alterations. Platelet-derived SCF was required for mPC3, but not RM1, tumor growth, while osteoblast-derived SCF played no role in tumor size in either cell line. While exogenous SCF induced proangiogenic protein secretion by RM1 and mPC3 prostate cancer cells, no significant changes in tumor angiogenesis were measured by immunohistochemistry. Like our previous studies, tumor-induced bone formation occurred in mice bearing RM1 or mPC3 neoplasms, demonstrated by bone histomorphometry. RM1 tumor-bearing osteoblast SCF knockout mice did not display tumor-induced bone formation. Bone stromal cell composition analysis by flow cytometry showed significant shifts in hematopoietic stem cell (HSC), mesenchymal stem cell (MSC), and osteoblast cell percentages in mice bearing RM1 or mPC3 tumors. There were no significant changes in the percentage of macrophages, osteoclasts, or osteocytes. Our study demonstrates that megakaryocyte/platelet-derived SCF regulates primary mPC3 tumor growth, while SCF originating from osteoblasts plays a role in bone marrow-derived progenitor cell composition and pre-metastatic niche formation. Further, we show that both the source of SCF and the genetic profile of prostate cancer determine the effects of SCF. Thus, targeting the SCF/CD117 signaling axis with tyrosine kinase inhibitors could affect primary prostate carcinomas or play a role in reducing bone metastasis dependent on the gene deletions or mutations driving the patients’ prostate cancer.

## Introduction

Skeletal metastasis is the leading cause of prostate cancer patient morbidity and mortality (1). Once the primary tumor has mobilized to the bone, the patient survival rate drops to less than 30% (2, 3). Most advanced prostate cancer patients experience complications with skeletal metastases such as bone pain, fractures, and spinal cord compression. Bone metastasis remains incurable; therefore, finding molecular targets to prevent and treat metastasis is urgently needed. The mechanism of prostate cancer metastasis to the bone is still unknown. However, active communication between the tumor and bone microenvironment is demonstrated by an increased bone formation that occurs prior to metastasis (4, 5).

The bone formation that occurs before the identification of measurable prostate cancer metastatic lesions results in the stimulation of osteoblasts and inhibition of osteoclasts. During homeostasis within the bone niche, there exists a balance between activation of the bone-forming cells, osteoblasts, and bone-resorbing cells, osteoclasts (6). Imbalances such as those occurring during prostate cancer progression result in altered bone metabolism, with prostate cancer stimulating an increase in bone formation. Platelets regulate this tumor-induced bone formation. Depletion of platelets in both xenograft and murine allograft models inhibited bone formation (7). Coupled with prostate cancer-induced osteoblast activation and bone formation, platelet production increases in response to tumor growth (8–12). Further, tumor-induced bone formation requires platelet secretion and can be regulated by several tumor-derived proteins sequestered in platelets (13, 14). Defining the platelet-derived proteins controlling communication between the primary tumor and the bone prior to metastasis is key to fighting metastatic disease.

Platelet-derived stem cell factor (SCF, Kit Ligand, Steel Factor) correlated with prostate cancer severity (15). SCF is expressed in both the primary tumor and bone metastases, while its sole ligand CD117/c-kit demonstrates increased expression in bone metastases compared with primary tumors (16). CD117 expression is also found on prostate cancer circulating tumor cells and is associated with a stem cell-like phenotype (15, 17). Thus, the SCF/CD117 signaling axis may play a role in platelet-regulated prostate cancer bone formation and metastatic spread.

Platelet SCF is likely packaged by megakaryocytes or may be sequestered from stromal cells in the bone microenvironment. Many bone marrow cell types express SCF, including perivascular cells, endothelial cells, pericytes, mesenchymal stem cells (MSCs), osteoblasts, and stromal cells (18–21). SCF in the bone microenvironment functions as a hematopoietic cytokine maintaining hematopoietic stem cell (HSC) proliferation and enhancing the differentiation of megakaryocytes and osteoclasts (22). This intercommunication between osteoblasts, osteoclasts, and megakaryocytes through SCF regulates HSC homing, bone formation, and platelet production. Thus, the platelet-derived SCF found in prostate cancer patients could originate from osteoblasts or megakaryocytes to control tumor-induced bone formation and prostate cancer spread.

To ascertain whether osteoblast or megakaryocyte/platelet-derived SCF played a role in prostate cancer progression, we depleted SCF in osteoblasts *via* the osteocalcin promoter and in megakaryocytes/platelets *via* the platelet factor 4 promoter using a conditional knockout murine model. Using two syngeneic tumor allografts, we examined the effect of SCF depletion on primary tumor growth, angiogenesis, and bone pre-metastatic niche formation. We found that SCF from megakaryocytes/platelets affects primary tumor growth, while SCF from osteoblasts plays a role in stem cell mobilization and pre-metastatic niche formation.

## Materials and Methods

### Cell Culture

Two murine prostate cancer cell lines, mPC3 and RM1, were used to study the effects of SCF. mPC3-luc (mPC3) murine prostate cancer cells were gifted by Dr. Zongbing You (Tulane University) and were generated by Dr. Zhenbang Chen (Meharry Medical College) (23, 24). The mPC3 cell line was generated from spontaneous tumors in probasin4-driven *Pten^-/-^;Trp53^-/-^* mice. These cells are grown in DMEM with 200 µg/mL hygromycin B and 10% FBS. RM1-luc-effly-eGFP (RM1) cells were gifted by Dr. Yusuke Shiozawa (Wake Forest School of Medicine). The parental RM1 cells (RRID: CVCL_B459) were obtained from ATCC prior to transfection with the luciferase/eGFP construct. The RM1 cells were initially derived from spontaneous prostate tumors that developed in *Ras* and *Myc* mice. These cells are grown in DMEM, 10% FBS, and penicillin/streptomycin (100 U/mL and 100 µg/mL, respectively). All cell lines are tested regularly for mycoplasma.

### 2D Confluence Assay

To examine proliferation, cell confluence was tracked by live-cell imaging. RM1 or mPC3 cells were seeded in a 96 well plate in complete media at 1,000 cells/well. At the time of seeding, 50 ng/mL of murine recombinant SCF (STEMCELL, #78064) was added. The cells were incubated at 37°C. Bright-field images were taken using the IncuCyte ZOOM live-cell imaging and analysis platform (Sartorius) in the Cell Engineering Shared Resource every 2 hours until confluent. Media was changed every three days. Percent confluence over 74 hours was analyzed using the IncuCyte Software (Version 2016A).

### Conditional Knockout Mouse Generation

Bone marrow SCF conditional knockout mice were generated in-house under approved Institutional Animal Care and Use Committee Protocols (A18-127, A15-194) at Wake Forest School of Medicine. To delete SCF (*Kitlg*) in megakaryocytes and platelets, the platelet factor 4 promoter (Cre-PF4) was used. To delete SCF in mature osteoblasts, the osteocalcin promoter (Cre-OC) was used. SCF floxed (RRID: IMSR_JAX:017861), Cre-PF4 (RRID: IMSR_JAX:008535), and Cre-OC (RRID: IMSR_JAX:019509) mice were purchased from Jackson Laboratory (Bar Harbor, MI) on a C57BL/6J background. Both conditional knockouts were generated by crossing *Cre^+/-^* mice with the SCF floxed mice to generate the F0 generation of *Cre^+/-^SCF^fl/^* ^-^. This F0 generation was again crossed with *SCF^fl/fl^* to generate the F1 *Cre^+/-^SCF^fl/fl^*. Finally, to generate our knockout models, F1 was intercrossed to generate the *Cre-PF4^-/-^;SCF^fl/fl^* (PLT-WT), *Cre-OC^-/-^;SCF^fl/f^* ^l^ (OB-WT), *Cre-PF4^+/-^;SCF^fl/fl^* (PLTΔSCF), and *Cre-OC^+/-^;SCF^fl/fl^* (OBΔSCF) mice.

### Tumor Growth

Male, 8-12-week-old knockout mice were bred and housed in the animal facilities at Wake Forest School of Medicine, fed a standard diet, and were on a standard light/dark cycle. All animal studies were approved by the Institutional Animal Care and Use Committee (Protocols A18-127, A15-221) at Wake Forest School of Medicine. Mice were anesthetized with isoflurane, and RM1 (4x10^5^ cells) or mPC3 cells (1x10^6^ cells) were injected subcutaneously on day 0. On day 11, mice were intraperitoneally injected with luciferin (150 mg/kg) to visualize tumor luciferase signal and then imaged using Perkin Elmer *In Vitro* Imaging System (IVIS) maintained by the Cell Engineering Shared Resource. Average radiance was analyzed using Living Image Software (Perkin Elmer). Tumors were allowed to grow for 12 days before sacrifice, and tumor weight and dimensions were measured. Tumor volume was calculated from caliper measurements using V = (W^2^ × L)/2 as the formula (25)

### Angiogenesis Protein Array

To measure the secretion of angiogenesis-related proteins, conditioned media was collected from prostate cancer cells treated with SCF. RM1 or mPC3 cells were grown on 10 cm tissue culture dishes and incubated at 37°C until they reached 70-80% confluence. The cells were then washed with serum-free media and treated with 50 ng/mL murine recombinant SCF for 24 hours. The cell culture supernatant was collected and centrifuged at 300 g for 10 min to remove cell debris. The conditioned media was stored at -80°C until further use. The Proteome Profiler Mouse Angiogenesis Array (R&D Systems, RRID: AB_1655573) was used in accordance with the manufacturer’s protocol with 700 µL of thawed supernatant. The array was analyzed by densitometry using Bio-Rad ImageLab. Proteins were normalized and compared to cells grown without SCF to calculate fold-change.

### Hindlimb and Tumor Tissue Processing

After 12 days, mice were humanely euthanized, and tumors and long bones were collected in 10% neutral, buffered formalin or PBS. After fixation, hindlimbs were cleaned by removing skin and muscle around the tibia and femur and decalcified in 14% neutral buffered EDTA for 2-3 weeks or until bones became soft. Tumors were fixed for 24-48 hours in 10% neutral, buffered formalin. All tissues were processed and embedded in-house using the following protocol. Dehydration from 50%-100% ethanol at 1 hour each was followed by two incubations in xylene for 1 hour each and two incubations in paraffin for 6 hours each. Tissues were then embedded in paraffin and sectioned at a thickness of 5 µm onto charged slides.

### Tumor Immunohistochemistry

To assess angiogenesis, tumors were stained for new vessel formation (CD31) and smooth muscle cell recruitment (αSMA). Tumors were sectioned and baked at 58°C for 1 hour. Antigen unmasking was performed by heat-induced epitope retrieval using 0.05% citraconic anhydride solution (PH 7.4) for 45 minutes at 98°C. Samples were blocked with 1% BSA for 30 minutes at room temperature then incubated with antibodies against CD31 (1:300, Abcam, RRID: AB_726362) or αSMA (1:2000, Abcam, RRID: AB_2223021) overnight at 4°C. The sections were visualized with ImmPACT NovaRED (HRP) Substrate (Vector Laboratories) and counterstained with hematoxylin Gill Method 1 (Fisher Scientific). Slides were scanned at 20X with a Hamamatsu Photonics Nanozoomer Slide Scanner in the Virtual Pathology Core. Visiopharm digital pathology analysis software (Version 2020.08, Visiopharm, RRID: SCR_021711) and custom-designed applications were used to quantify the percent of positive immunostained areas. A region of interest was drawn around the tissue, the area of the positive staining was identified and measured within the region of interest, and the ratios of the positive staining area to the total area were calculated.

### TRAP Staining and Bone Histomorphometry Analysis

To assess the bone structure and osteoclast presence, long bone sections were stained for tartrate-resistant acid phosphatase (TRAP), and bone histomorphometry was analyzed. TRAP buffer was prepared in-house with 0.1 M acetate buffer, 0.3 M sodium tartrate, 10 mg/mL naphthol solution, triton x-100, and Fast Red Violet at pH 5.0. Sections were baked onto positively charged slides for 1 hour at 58°C. Slides were deparaffinized 3x in xylene and rehydrated from 100%-70% ethanol with a final wash in water. Slides were incubated in TRAP solution at 37°C for 1 hour. Slides were rinsed with water, counterstained with hematoxylin for 1 min, and washed with deionized water before dehydrating. To dehydrate, slides were incubated in increasing ethanol (70%-100%) then incubated in xylene 3x for 2 min. Images were scanned at 20X using the Hamamatsu Photonics Nanozoomer Slide Scanner in the Virtual Pathology Core and analyzed in-house using the BioQuant Osteo software (BioQuant Osteo 2016 v16.1.60, RRID: SCR_016423). Images were analyzed by drawing a region of interest in the diaphysis starting 150 microns distal to the growth plate of the tibia at 1500 µm length by width. Measurements generated using the software were Bone Volume normalized by Tissue Volume (BV/TV, Bone Fraction, %), Bone Surface normalized by Bone Volume (BS/BV, 1/mm), Number of Osteoclasts per millimeter of Bone Surface (Oc.S/BS). Trabecular Thickness (Tb.Th) was calculated using Tb.Th =2/(BV/BS)*1000.

### Bone Stromal Cell Flow Cytometry

To measure bone marrow cell composition changes, bone stromal and bone-residing cells were isolated and profiled by flow cytometry (26). Hind limbs were collected from mice after sacrifice and marrow extruded in PBS to collect HSCs, MSCs, and macrophages. The remaining bone underwent partial collagenase digestion (1 mg/mL) to release bone-residing cells: osteoblasts, osteoclasts, and osteocytes. Cells were resuspended in fluorescence-activated cell sorting buffer (BM-FACS buffer) and blocked with FcR mouse blocking reagent (2 µL/1x10^7^ cells, Miltenyi Biotec, 130-092-575). BM-FACS buffer was composed of 3% bovine serum albumin (BSA, A3059-100g), 2 mM EDTA (Fisher, S311-100), and 10 mM HEPES (Gibco,15-630-080) in 1x PBS. The cells were then stained with ZombieAqua™ live/dead stain (1:1000, BioLegend, 423102). The sample was divided into 1x10^6^ cells/100 µL and stained with the appropriate cell identification antibody mix described below using the antibodies listed in **Supplementary Table 1**. Bone marrow was analyzed for HSCs (CD34+, CD45+, Sca1+) MSCs (Sca1+, CD146+, CD29+, CD90+), Osteoblasts (Alkaline Phosphatase+, CD90+), Osteoclasts (CD11b-, CD115+, CD68+, RANKL+), Osteocytes (GP38+, SPARC+), and Macrophages (CD11b+, CD115+, CD68+, RANKL-). Each sample was then fixed in 1% methanol-free paraformaldehyde (Polysciences INC, 04018-1) in PBS. Samples were analyzed using the BD FACSCanto™ II (BD Biosciences), maintained by the Flow Cytometry Shared Resource, and FlowJo analysis software (RRID: SCR_008520).

### Statistical Analysis

Comparisons of means among more than two groups were analyzed using analysis of variance (ANOVA) with Tukey post-testing. Between two groups, analysis was performed using a two-tailed unpaired Student’s t-test. For proliferation rates, a two-tailed nonparametric t-test was performed with the Mann-Whitney test to compare ranks. Data were analyzed using Prism 9 (GraphPad Software, RRID: SCR_002798). Error bars represent the experimental standard error of the mean (SEM). * represents p<0.05, ** represents p<0.005, *** represents p<0.0005, and **** represents p<0.0001.

## Results

### SCF Has No Effect on *In Vitro* Proliferation

**Figure 1:**
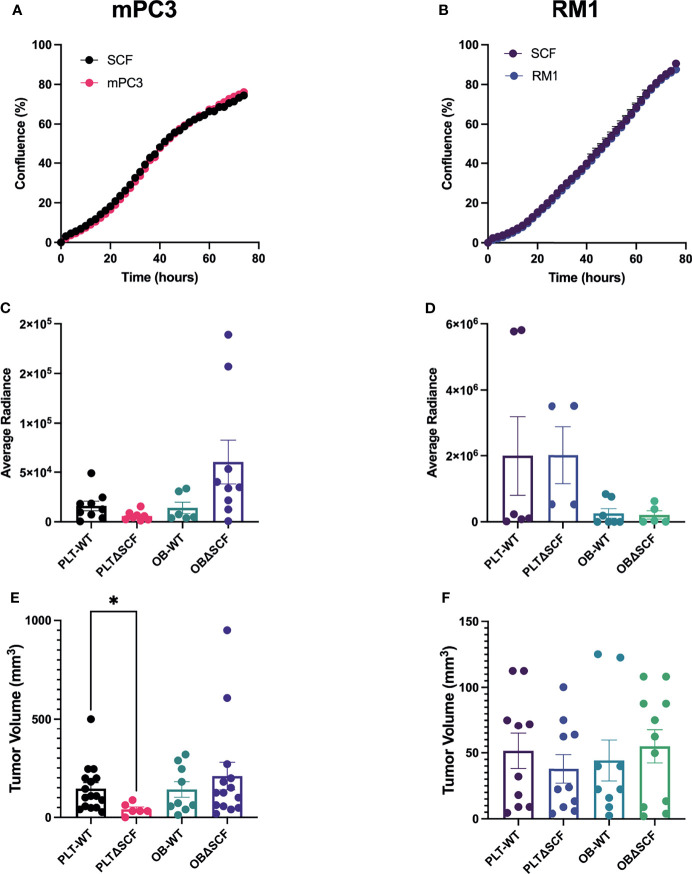
Platelet deletion of SCF reduces mPC3 tumor growth. mPC3 **(A)** and RM1 cells **(B)** were treated with 50 ng/mL of SCF, and proliferation measured over 74 hours represented as mean percent confluence ± SEM (n=3). **(C–F)** mPC3 and RM1 cells were injected subcutaneously into PLT-WT, PLTΔSCF, OB-WT, or OBΔSCF mice. Tumors were allowed to grow for 12 days and imaged *via* IVIS on day 11 for average radiance **(C, D)**. Tumor volume **(E, F)** and average radiance are represented by mean ± SEM (n = 4-9). * represents p < 0.05 by unpaired t-test between mice of the same background.

Our prior study indicated that exogenous SCF induced proliferation of human prostate cancer cells expressing the tyrosine kinase receptor CD117 (17). Like human prostate cancer cells, both the *Ras/Myc* overexpressing RM1 and the *Pten^-/-^;Trp53^-/-^* mPC3 murine prostate cancer cell lines contain a subpopulation of CD117 expressing cells: 10-15% and 20-40%, respectively (data not shown). Thus, we performed live-cell imaging-based proliferation assays to assess the effect of SCF on prostate cancer growth. There was no change in percent confluence after treatment with 50 ng/mL of SCF for either mPC3-luc (mPC3) or RM1-luc-effly-eGFP (RM1) cells over 60 hours (**Figures 1A, B**). Thus, exogenous SCF did not affect the proliferation of the murine prostate cancer cells *in vitro*.

### PLTΔSCF mPC3-luc Tumor-Bearing Mice Have Decreased Tumor Volume

To examine the effects of bone marrow-derived SCF on primary tumor growth, we generated conditional SCF knockout mice with SCF deleted in megakaryocytes and platelets (PLTΔSCF) or mature osteoblasts (OBΔSCF) using a Cre-lox system. In these mice, both the membrane and soluble form of SCF are deleted from the target cells. To implant primary tumors, syngeneic mPC3 or RM1 cells were injected subcutaneously into the left flank of control (PLT-WT, OB-WT), megakaryocyte and platelet SCF deleted PLTΔSCF, or mature osteoblast SCF deficient OBΔSCF mice (**Figures 1C–F**). The tumors were allowed to grow for 12 days post-injection. Since both cell lines expressed luciferase, bioluminescent imaging was performed one day before sacrifice. No significant change was measured for the average radiance for mPC3 or RM1 tumors in both genotypes (**Figures 1C, D**; **Supplementary Figure 1**). However, radiance for mPC3 tumors was 2.7-fold higher in PLT-WT tumors compared with PLTΔSCF tumors (p=0.08) and 4.3-fold higher in OBΔSCF tumors compared with OB-WT tumors (p=0.11). Tumors were then collected on day 12, and tumor volume was calculated to determine the effect of SCF on primary tumor growth. Deletion of SCF in platelets and megakaryocytes (PLTΔSCF) caused a significant decrease (p <0.05) in mPC3 tumor volume (**Figure 1E**) compared to PLT-WT. The average mPC3 tumor volume for PLTΔSCF was decreased 3.6-fold compared with PLT-WT tumors (41.04 mm^3^ and 145.85 mm^3^, respectively). In fact, many of the mPC3 tumors in PLTΔSCF mice did not develop. No difference was measured in tumor volume for mPC3 tumors between OBΔSCF and OB-WT mice (**Figure 1E**). The average mPC3 tumor volume for OBΔSCF was 209.25 mm^3^ compared to OB-WT at 141.55 mm^3^. As well, there was no difference in tumor volume for RM1 tumor-bearing mice for PLTΔSCF or OBΔSCF compared with their WT controls (**Figure 1F**). These data demonstrate that platelet-derived SCF was important for mPC3 tumor growth.

### SCF Causes an Increase in Proangiogenic Protein Secretion

**Figure 2:**
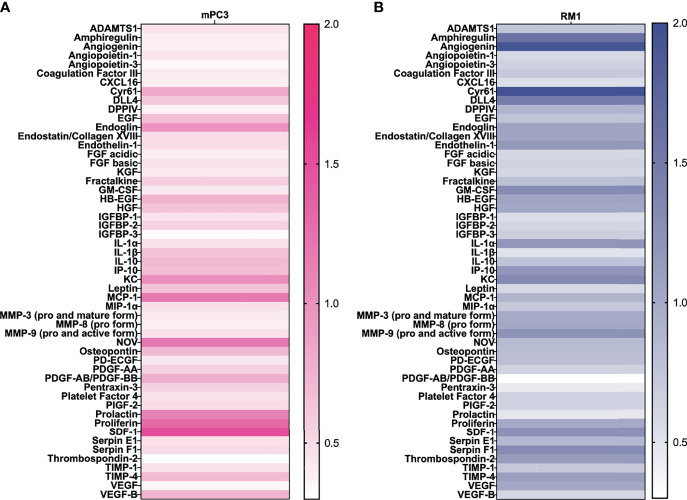
SCF causes an increase in angiogenic factors in vitro. mPC3 **(A)** and RM1 **(B)** cells were treated with 50 ng/mL SCF for 24 hours. Conditioned media was collected and analyzed using an angiogenesis protein array. Densitometry values were normalized to conditioned media from untreated cells.

SCF is known to stimulate angiogenesis (27), an essential process for tumor growth. To determine which proangiogenic factors SCF regulated, prostate cancer conditioned media were analyzed using an angiogenesis protein profiler array after treatment with 50 ng/mL SCF for 24 hours. For mPC3 cells, four angiogenic proteins were increased more than 1.2-fold after SCF treatment (**Figure 2A**) compared to the untreated control group. Increased proangiogenic proteins were monocyte chemoattractant protein-1 (1.2-fold increase), nephroblastoma overexpressed (1.2-fold increase), proliferin (1.3-fold increase), and stromal cell-derived factor-1/CXCL12 (SDF-1; 1.5-fold increase). The other angiogenic proteins had unchanged or decreased expression compared to mPC3 cells without treatment. SCF stimulated a 1.2-fold or higher release of 12 angiogenic proteins from RM1 cells (**Figure 2B**), which included amphiregulin (1.6-fold increase), angiogenin (1.9-fold increase), cysteine-rich angiogenic inducer 61 (2.0-fold increase), delta-like canonical notch ligand 4 (1.5-fold increase), endothelin-1 (1.2-fold increase), granulocyte-macrophage colony-stimulating factor (1.3-fold increase), interleukin-1 alpha (1.2-fold increase), CXCL10/IP-10 (1.2-fold increase), CXCL1/KC (1.3-fold increase), matrix metalloproteinase 9 (1.2-fold increase), SDF-1 (1.3-fold increase), and Serpin F1 (1.3-fold increase). The other proangiogenic proteins had decreased expression or no change in RM1 cells treated with SCF. These data demonstrate that SCF induces different proangiogenic signaling pathways in RM1 and mPC3 cells, with only SDF-1 increasing in both cell lines.

### Bone-Derived SCF Did Not Affect Tumor Angiogenesis

**Figure 3:**
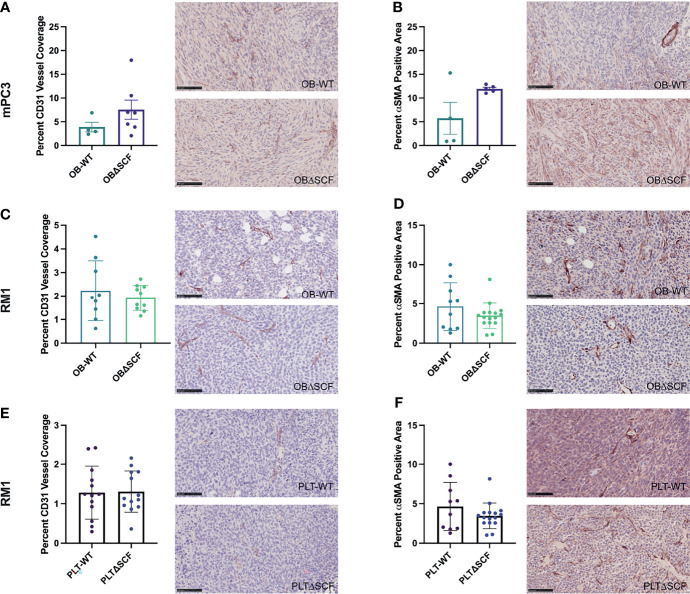
Bone marrow derived-SCF loss does not affect tumor angiogenesis. mPC3 **(A, B)** and RM1 **(C–F)** tumors from PLT-WT, PLTΔSCF, OB-WT, or OBΔSCF mice were collected and stained for CD31 **(A, C, E)** or α-SMA **(B, D, F)**. CD31 was measured as percent CD31 vessel coverage, and α-SMA was calculated as percent α-SMA positive area and represented as mean ± SEM (n = 4-9). Scale bars represent 100 μm.

To examine angiogenesis and vascular maturation, immunohistochemistry was performed on tumor tissues. Staining for the endothelial cell marker CD31 was performed to measure blood vessel coverage in tumors, while smooth muscle actin (αSMA) staining was used to differentiate mature blood vessels (28). Tumors from PLTΔSCF mice injected with mPC3 cells were too small for downstream analysis, and thus, we were unable to compare angiogenesis in mPC3 tumors from PLTΔSCF with PLT-WT tumors. While not significant, OBΔSCF mPC3 tumors tended to have increased percentage of αSMA positive cells (2.1-fold, p=0.077) and CD31-positive vessel coverage (1.9-fold, p=0.22) compared with OB-WT mPC3 tumors (**Figures 3A, B**). These data align with the higher tumor volumes seen in **Figure 1** for mPC3 tumors, although the effects did not reach significance. For mice injected with RM1 tumors, PLTΔSCF and OBΔSCF had no significant difference in percent αSMA positive area or CD31-positive blood vessel coverage compared to the PLT-WT and OB-WT tumors (**Figures 3C–F**). Thus, SCF deletion had no significant effect on tumor angiogenesis.

### Bone Marrow Deletion of SCF Did Not Affect the Bone Structure in Tumor-Bearing Mice

**Figure 4:**
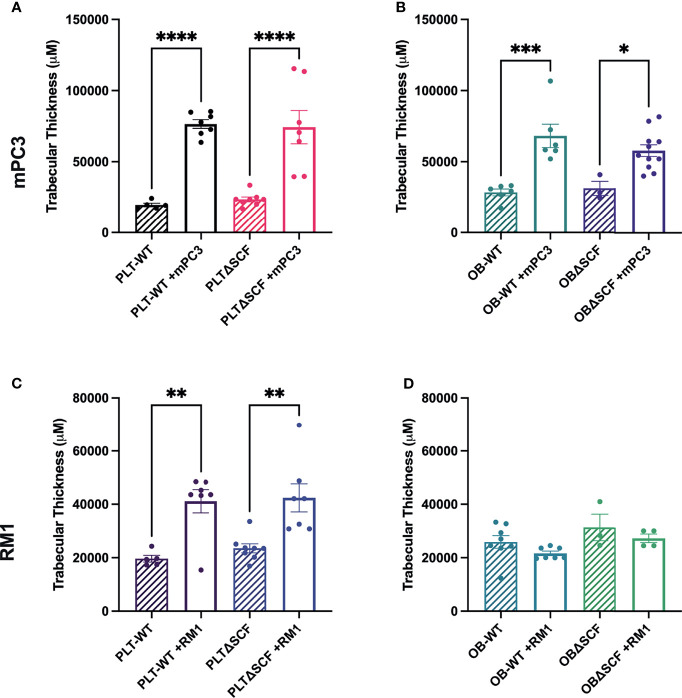
Osteoblast-derived SCF regulates RM1 tumor-induced bone formation. Tibiae from mPC3 **(A, B)** and RM1 **(C–F)** tumor-injected mice were compared with tibiae from non-tumor bearing control PLT-WT and PLTΔSCF **(A, C)** or OB-WT and OBΔSCF **(B, D)** mice. Trabecular thickness measured by bone histomorphometry is represented as mean ± SEM (n=4-9). * represents p < 0.05, ** represents p < 0.005, *** represents p < 0.0005 and **** represents p < 0.0001 by one-way ANOVA.

Our prior studies demonstrated that subcutaneous RM1 tumor growth induced bone formation and that platelets governed this pre-metastatic communication with the bone microenvironment (7, 29). To examine whether deletion of bone marrow-derived SCF would alter the bone microenvironment, bone sections from tumor-bearing mice were stained for tartrate-resistant acid phosphatase (TRAP) positive osteoclasts to determine osteoclast number and measure bone histomorphometry. mPC3 tumor growth stimulated bone formation in PLTΔSCF (3.2-fold, p<0.0001), PLT-WT (3.9-fold, p<0.0001), OBΔSCF (1.8-fold, p=0.036), and OB-WT (2.4-fold, p=0.0003) mice (**Figures 4A, B**). Interestingly, RM1 tumor growth induced bone formation in PLTΔSCF (1.8-fold, p=0.005) and PLT-WT (2.1-fold, p=0.005) but not in OBΔSCF or OB-WT mice (**Figures 4C, D**). Further, there was no difference in tumor-induced bone formation with SCF deletion in either osteoblasts or megakaryocytes and platelets. Neither bone fraction (BV/TV), osteoclast surface fraction (OC.s/BS), nor trabecular thickness (Tb.Th) was altered between groups (**Table 1**). No significant differences in bone histomorphometry were seen between PLTΔSCF and PLT-WT or OBΔSCF and OB-WT in mice without tumors (**Figure 4**). Thus, tumor-induced bone formation still occurred in most mice and was not affected by SCF deletion.

**Table 1:** Average relative search volume (RSV) and information prevalence from Google search and trends.

Parameter	WT Mean	SEM	ΔSCF Mean	SEM	p-value	n
***mPC3 PLT***						
*BV/TV*	0.21	0.034	0.22	0.031	0.83	7
*OC.S/BS*	0.14	0.15	0.19	0.34	0.20	7
*Tb.Th*	76,582	3,087	74,392	11,697	0.86	7
***mPC3 OB***						
*BV/TV*	0.20	0.031	0.25	0.026	0.94	6-11
*OC.S/BS*	0.19	0.031	0.11	0.012	0.14	6-11
*Tb.Th*	82,540	8,182	62,179	4,135	0.22	6-11
***RM1 PLT***						
*BV/TV*	0.31	0.025	0.28	0.029	0.44	8-10
*OC.S/BS*	0.14	0.031	0.12	0.022	0.70	8-10
*Tb.Th*	41,126	4,366	42,411	5,240	0.85	8-10
***RM1 OB***						
*BV/TV*	0.22	0.024	0.28	0.037	0.24	8-10
*OC.S/BS*	0.19	0.043	0.16	0.021	0.86	8-10
*Tb.Th*	21,154	1,430	24,264	1,258	0.26	8-10

Bone fraction (BV/TV); Osteoclast surface fraction (OC.S/BS); Trabecular thickness (Tb.Th).

### Osteoblast-Derived SCF Plays a Role in Bone Stem Cell Populations

Alterations in the bone structure result in changes in the composition of the bone microenvironment with shifts in the numbers of osteoclasts and osteoblasts. Further, the bone marrow HSC niche is the colonization site for disseminated cancer cells in murine bone (30, 31). These cells then compete for space in the bone marrow with metastatic lesions, causing a decrease in the HSC population in the bone (32). Once these cancer cells have disseminated and metastasized to the bone, osteoblasts act as an anchor and play a role in dormancy (33, 34). Thus, the bone niche cellular composition plays a vital role in tumor metastasis and creating the pre-metastatic niche.

**Figure 5:**
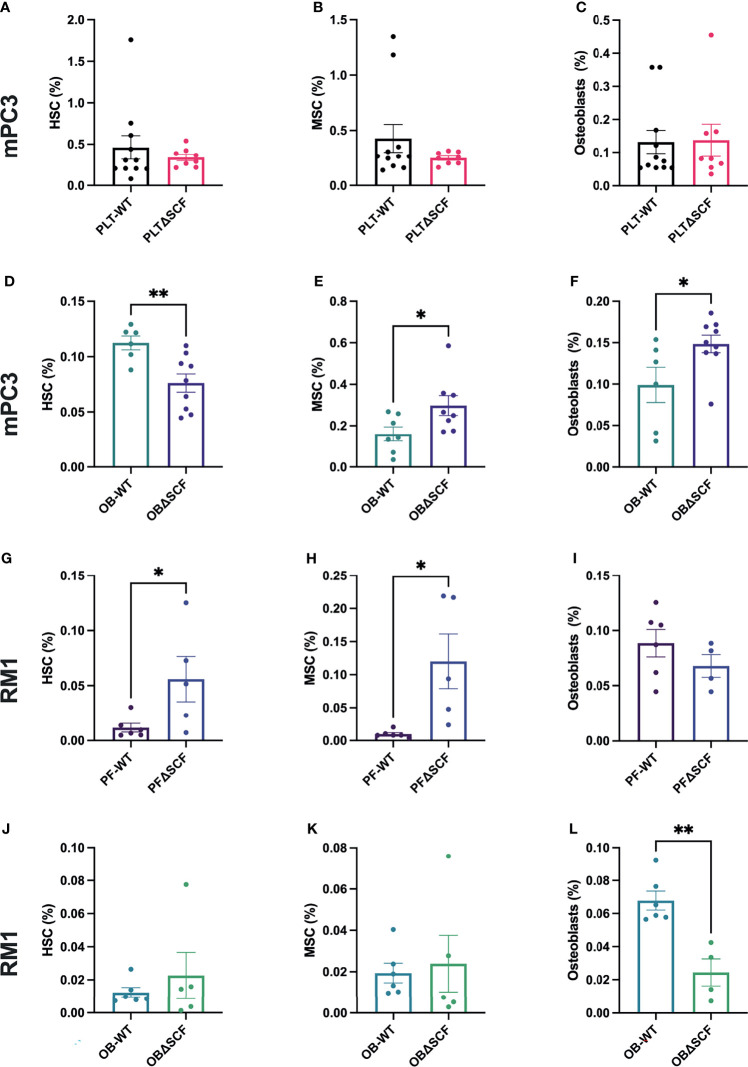
SCF mediated alterations of the bone niche composition. Tibiae were isolated from PLT-WT, PLTΔSCF **(A–C, H–J)**, OB-WT, or OBΔSCF **(D–F, K–M)** mice after tumor implantation with mPC3 **(A–F)** or RM1 **(H–M)** prostate cancer cells. Bone marrow was isolated and stained for HSCs **(A, D, H, K)**, MSCs **(B, E, I, L)**, and osteoblasts **(C, F, J, M)**. Flow cytometry was performed to calculate the percent cell population represented as mean ± SEM (n = 6-9). * represents p < 0.05 and ** represents p < 0.005 by unpaired t-test.

To determine how SCF from osteoblasts or platelets and megakaryocytes affects the pre-metastatic bone niche, bone marrow and bone-residing cells after partial collagenase digestion were collected from tumor-bearing mice, and flow cytometry was performed to analyze different bone cell progenitor and stromal cell populations. We used specific cell surface markers to differentiate HSCs, MSCs, macrophages, osteoblasts, osteoclasts, and osteocyte populations. While not significant, macrophage (2.0-fold, p=0.056) and osteoclast (2.3-fold, p=0.12) populations were decreased in PLTΔSCF compared to PLT-WT mPC3 tumor-bearing mice (**Supplementary Figures 2A, B**). No difference was seen in HSCs, MSCs, osteoblasts, or osteocyte populations in these mice (**Figures 5A–C**; **Supplementary Figure 2C**). Conversely, osteoblast-derived SCF played a significant role in mPC3 tumor-bearing bone progenitor cell populations. HSC numbers were significantly decreased (2.6-fold decrease, p=0.007) in OBΔSCF mPC3 tumor-bearing mice compared to OB-WT (**Figure 5D**). In contrast, the MSC population significantly increased (1.9-fold, p=0.04) in the OBΔSCF compared to the OB-WT mice (**Figure 5E**). In addition, OBΔSCF mPC3 tumor-bearing mice had a significant increase (1.5-fold increase, p=0.038) in osteoblast numbers (**Figure 5F**) compared to OB-WT tumor-bearing mice. While not significant, macrophages tended to be increased (1.9-fold, p=0.095), and osteoclasts (3.6-fold, p=0.087) were decreased in OBΔSCF mPC3 tumor-bearing mice compared to OB-WT. There was no difference in the osteocyte population (**Supplementary Figures 2D–F**). These data indicated that for mPC3 tumors, osteoblast-derived SCF might alter the colonization and dormancy niches in the bone microenvironment for metastatic cells.

The effects of bone marrow-derived SCF were different for RM1 tumors. PLTΔSCF RM1 tumor-bearing mice showed a significant increase in HSC (4.8-fold increase, p=0.046) and MSC (12.3-fold increase, p=0.02) populations as shown in **Figures 5H, I**. There was no difference in osteoblast, macrophage, osteoclasts, or osteocyte populations (**Figure 5J**, **Supplementary Figures 2H–J**). OBΔSCF RM1 tumor-bearing mice demonstrated a similar, but not significant, increase in HSC (1.8-fold, p=0.45) and MSC (1.2-fold, p=0.74) numbers (**Figures 5K, L**). However, osteoblast numbers were significantly decreased in OBΔSCF mice compared to OB-WT (2.8-fold, p=0.002, **Figure 5M**), which directly contrasts the data seen for mPC3 tumors. OBΔSCF RM1 tumor-bearing mice had a non-significant increase in osteoclast numbers (4.5-fold, p=0.087) but no difference in macrophage or osteocyte populations compared with OB-WT (**Supplementary Figures 2K–M**). There was no significant difference in bone cell populations in mice without tumors between PLTΔSCF and PLT-WT or OBΔSCF and OB-WT (data not shown). Thus, the effects on the bone microenvironment progenitor cell population and metastatic niche composition can be altered by bone marrow-derived SCF, but the result is dependent on the tumor cell line studied.

## Discussion

This study aimed to characterize the role of bone marrow-derived SCF in primary tumor growth, angiogenesis, and the pre-metastatic bone niche. We found that the source of SCF and the prostate cancer’s genetic background both played a role in disease progression. SCF originating from megakaryocytes and platelets caused significantly decreased mPC3 tumor growth, while there was no effect in RM1 tumors. Osteoblast-derived SCF did not affect tumor growth. Angiogenesis and tumor-induced bone formation were not affected by SCF deletion in either genetic background. However, there were significant shifts in bone marrow composition. Osteoblast-derived SCF loss decreased HSCs and increased MSCs and osteoblasts in mPC3 tumor-bearing mice, while platelet deletion had no effect. For RM1 tumor-bearing mice, platelet depletion of SCF increased HSC and MSC progenitor cell populations with the loss of SCF in osteoblasts resulting in reduced osteoblast numbers. Thus, our data demonstrate that megakaryocyte and platelet-derived SCF regulates primary mPC3 tumor growth, while SCF originating from osteoblasts plays a role in bone marrow progenitor cell mobilization and pre-metastatic niche formation.

The role of SCF in prostate cancer tumor growth differed based on the model tested. Platelet and megakaryocyte depletion of SCF dramatically reduced mPC3 tumor volume, which could be due to alterations in proliferation, angiogenesis, or other cell survival pathways. Proliferation *in vitro* was not affected by exogenous SCF for either the mPC3 or RM1 cells, indicating that this mechanism is unlikely to be the main reason for reduced mPC3 tumor growth. RM1 tumors in platelet SCF depleted mice did not have significantly reduced tumor size. This may be due to fewer CD117 receptors on the RM1 cells. The mPC3 cells have a higher CD117 subpopulation, so they may be more reliant on CD117 activation for tumor growth and angiogenesis. Our prior data demonstrate that CD117 expression on prostate cancer stem-like cells is associated with tumor initiation (17). The loss of CD117 activation in a subpopulation of mPC3 cells could also reduce tumor formation and growth in mice after platelet SCF depletion. The effect of SCF on other pathways supporting mPC3 tumor growth, including hypoxia and apoptosis resistance warrants further study.

Platelets regulate angiogenesis (9, 34–37), and SCF binding to CD117 activates a signaling cascade stimulating angiogenesis (38, 39). Due to the size of mPC3 tumors after platelet SCF deletion, blood vessel formation could not be examined and remains a potential mechanism by which platelet SCF controls mPC3 tumor growth. Treatment of prostate cancer cells with SCF *in vitro* resulted in the secretion of proangiogenic proteins that may be required for blood vessel development or stabilization but only SDF-1 was common between the two prostate cancer cell lines. Tumor-derived SDF-1 is upregulated in platelets of RM1 tumor-bearing mice (13) and increased circulating SDF-1 is associated with enhanced homing of CXCR4-positive bone marrow-derived progenitor cells to tumors driving angiogenesis (14). Thus, reduced SDF-1 secretion by prostate cancer cells after depletion of platelet SCF could result in diminished mPC3 tumor growth due to inhibition of angiogenesis through effects on the SDF-1/CXCR4 pathway.

Osteoblast secretion of SCF was not necessary for either RM1 or mPC3 tumor growth or angiogenesis. This lack of response is not surprising as SCF deletion occurs in the bone microenvironment distal to the primary tumor. Platelets circulating between the osteoblast microenvironment and primary tumors would be exposed to many potential sources of SCF, including endothelial cells (19–21). In the bone microenvironment, osteoblast SCF regulates megakaryocyte function (40, 41), and a negative feedback loop could result in an upregulation of megakaryocyte SCF production (42). This could increase the amount of SCF in platelets in mice with osteoblast SCF deletion.

Bone stromal cells such as osteoblasts, osteoclasts, MSCs, HSCs, and megakaryocytes can accelerate or impede skeletal metastasis (43, 44). The ratio and activation status of osteoblasts and osteoclasts directly affect bone remodeling and the overall pre-metastatic niche. Prostate cancer can cause an osteoblastic, osteolytic, or mixed phenotype before and after a metastatic lesion has formed (7) and prostate cancer is more likely to metastasize during bone remodeling (45). Like our prior studies, both RM1 and mPC3 tumor growth induced bone formation. However, this was not affected by either megakaryocyte and platelet or osteoblast SCF deletion. Beyond changes in the bone structure, tumors can cause alterations in the bone marrow cell composition. For example, tumor growth stimulates bone marrow-derived progenitor cell mobilization (14, 46–48). Osteoblast deletion of SCF reduced hematopoietic lineage cells (HSCs and osteoclasts) and increased mesenchymal lineage cells (MSCs and osteoblasts) in mice bearing mPC3 tumors. The MSC population was also increased in mice after osteoblast deletion of SCF and in RM1-bearing megakaryocyte and platelet-depleted SCF mice. SCF does not affect the proliferation of MSCs but increases expression of adhesion molecules and matrix metalloproteinases controlling migration (49). Thus, the loss of SCF in the bone microenvironment may be preventing MSC mobilization. In contrast, osteoblast percentages depended on the prostate cancer’s genetic background. mPC3 tumor growth increased osteoblast numbers, while RM1 tumor growth reduced osteoblast numbers in mice with osteoblast deletion of SCF. This alteration in osteoblast numbers could affect the dormancy of disseminated prostate cancer cells. In addition, quiescent, bone-lining osteoblasts secrete undetectable SCF, while activated bone-forming osteoblasts along the mineralization front have higher SCF production (50). Thus, tumor-induced bone formation could increase SCF production through osteoblast activation which may have subsequent effects on prostate cancer cell homing to the bone microenvironment.

Counterbalancing the mesenchymal lineage, the hematopoietic lineage cells were also altered in response to tumor growth. Mice bearing RM1 tumors demonstrated an increase in HSC numbers in the bone marrow, although this was only statistically significant with megakaryocyte and platelet depletion of SCF. Conversely, osteoblast deletion of SCF reduced HSC numbers in mPC3 tumor-bearing mice. These alterations in the HSC counts could either be through altered HSC mobilization or proliferation. In prior studies, the reductions in HSCs seen with perivascular and mesenchymal SCF deletion were not due to proliferation differences (51), indicating that proliferation is likely not the mechanism controlling HSC populations in our SCF deletion models. Thus, the reduction in HSCs may be due to altered mobilization into the circulation. Studies suggest that membrane-bound SCF in the bone is an important adhesion molecule for HSCs and a decrease in SCF causes an increase in HSC mobilization (52). Further studies demonstrated that the effect of SCF on HSCs is dependent on the source. SCF deletion in perivascular stromal cells or mesenchymal lineage cells (osteocytes, chondrocytes, and adipocytes) led to reduced HSC numbers in the bone marrow (53). While studies show that osteoblast-derived SCF does not affect HSCs (21, 51), the differentiation status of the osteoblast may alter its crosstalk with HSCs. More differentiated, bone-forming osteoblasts increase HSC renewal through membrane-bound SCF and cell-cell interaction, while less differentiated, more mesenchymal osteoblasts secrete more cytokines capable of signaling to HSCs (50, 54). Our genetic deletion removed both the membrane and soluble forms of SCF and only in terminally differentiated osteoblasts, unlike prior studies that deleted SCF earlier in osteoblast differentiation. In addition, membrane-bound SCF binding to CD117 on bone stromal cells stimulates megakaryocyte DNA synthesis and proliferation (55). Approximately 20% of HSCs can be found directly adjacent to megakaryocytes along bone marrow sinusoids, with 50% of HSCs being within two cell diameters of megakaryocytes (56, 57). Megakaryocyte depletion increases HSC proliferation and cell numbers (56). Further, platelet depletion induces membrane localization of SCF on megakaryocytes and stimulates nearby HSC proliferation (58). The number megakaryocytes increase with age leading to higher platelet counts (59) and higher numbers of HSCs in the bone marrow. Since most men develop prostate cancer at an advanced age, megakaryocyte and platelet SCF may play a more prominent role in older patients, which was not studied here. The effects of SCF loss on prostate cancer progression depended not only on the source of SCF but also on the genetic background of the prostate cancer cell lines.

Our study examined murine prostate cancer cell lines developed to mimic the common genetic mutations in prostate cancer patients with castration-resistant disease: *MYC, RAS, PTEN*, and *TP53*. The proto-oncogene *MYC* is expressed in approximately 40% of primary adenocarcinomas and 90% of metastases, with metastases often displaying gene amplification (60, 61). The tumor suppressor *TP53* is frequently mutated or deleted in cancers, with mutations in 8% of primary prostate adenocarcinomas and 47% of metastatic prostate cancers (62, 63). Deletions of *PTEN* are often associated with aggressive prostate cancer and can be found in up to 17% of primary prostate cancer patients and in 41% of metastatic cancers (60, 63). The oncogenes encoding the Ras protein are activated in many prostate cancers (up to 24%) and are associated with higher staged prostate carcinomas (60). Our data demonstrate that the genetic background of the cells played a significant role in the study outcomes. *Myc/Ras* co-activation is associated with prostate cancer bone metastasis in mice with a prostate-specific *Pten* deletion background and in patient bone biopsies (64). Further, *Myc/Ras* co-activation does not play a role in prostate cancer patient primary tumors. Thus, the lack of response to SCF depletion in RM1 (*Ras* and *Myc* co-activation) primary tumor growth and angiogenesis in our study is less surprising. Ras pathway activation stimulates angiogenesis in tumors (65), and thus, the overactivation of the Ras pathway may be why there were no significant differences in vessel formation in the RM1 tumors. In contrast, mPC3 (*Pten* and *Trp53* deletion) tumor growth was significantly reduced by platelet and megakaryocyte SCF loss and may increase with osteoblast SCF deletion. *TP53* and *PTEN* coalterations are found in 17% of localized prostate cancer and 16% of metastatic castration-sensitive prostate cancer, increasing to 56% for metastatic castration-resistant prostate cancers (66). Thus, the effects of SCF deletion on mPC3 tumor growth may be dependent on the source of SCF and the site of the tumor. The gene encoding SCF’s ligand CD117, *KIT*, is the most prevalently mutated gene in prostate cancer patients, in addition to *RAS* and *TP53*, and is associated with aggressive prostate cancer (67). Thus, SCF may play a greater role in prostate cancer colonization and engraftment in the bone microenvironment during metastasis which will be the subject of future studies.

In summary, we determined that SCF from megakaryocytes and platelets is important for primary tumor growth in mPC3 tumor-bearing mice. While in RM1 tumor-bearing mice, SCF from platelets affects HSC and MSC pre-metastatic niche populations. SCF from osteoblasts alters bone marrow progenitor cell composition and pre-metastatic niche formation for both RM1 and mPC3 tumor-bearing mice. We demonstrate that the origin of bone marrow-derived SCF and the genetic background of the prostate cancer have differential effects on primary growth and pre-metastatic niche formation. Thus, treating patients with tyrosine kinase inhibitors targeting the SCF/CD117 pathway requires consideration of the patient’s genetic profile. Further, the effects of SCF pathway intervention will likely differ based on the stage of the prostate cancer.

## Data Availability Statement

The raw data supporting the conclusions of this article will be made available by the authors, without undue reservation.

## Ethics Statement

The animal study was reviewed and approved by Wake Forest School of Medicine Institutional Animal Care and Use Committee.

## Author Contributions

Conceptualization: BK. Formal Analysis: BF and LS. Funding Acquisition: BK. Investigation: BF, LS, KH, CP, VS, and KL. Project Administration: BK. Supervision: BK. Visualization: BF. Writing – Original Draft: BF. Writing – Review and Editing: BF, LS, KH, CP, VS, KL, and BK.

## Funding

This work was supported by research funding from the National Cancer Institute at the National Institutes of Health (R00 CA175291 to BK). The use of the core services: Cell Engineering Shared Resource and Flow Cytometry Shared Resource, were partially subsidized by the Wake Forest Baptist Comprehensive Cancer Center Shared Resources grant (NIH/NCI CCSG P30 CA012197). This research was additionally supported by a Wake Forest CTSI grant (NIH/NCATS UL1 TR001420) which supports the Virtual Pathology Core.

## Conflict of Interest

The authors declare that the research was conducted in the absence of any commercial or financial relationships that could be construed as a potential conflict of interest.

## Publisher’s Note

All claims expressed in this article are solely those of the authors and do not necessarily represent those of their affiliated organizations, or those of the publisher, the editors and the reviewers. Any product that may be evaluated in this article, or claim that may be made by its manufacturer, is not guaranteed or endorsed by the publisher.
