# Supplementary material for: Bone Marrow-Derived Stem Cell Factor Regulates Prostate Cancer-Induced Shifts in Pre-Metastatic Niche Composition
Source: Front Oncol. 2022 Apr 19;12:855188. doi: 10.3389/fonc.2022.855188 (PMC9063312; doi:10.3389/fonc.2022.855188)
Supplement: Supplementary file 1 [file DataSheet_1.docx]

Supplementary Material

**1 Supplementary Table 1. Antibodies for Multi-Fluorophore Flow Cytometric Analysis of Bone Stromal Cells**

| **Antibody** | **Volume /Test** | **Conjugation** | **Isotype Control** | **Company** | **RRID** |
| --- | --- | --- | --- | --- | --- |
| CD34 | 1 µL | PE | PE Rat IgG2a, k | BioLegend | AB_2629648 |
| CD45 | 1 µL | APC | APC Rat IgG2b,k | BioLegend | AB_2563535 |
| Sca1 | 1 µL | FITC | FITC Rat IgG2a, k | BioLegend | AB_313343 |
| CD146 | 0.3 µL | PE | PE Rat IgG2a,k | BioLegend | AB_2143527 |
| CD29 | 0.5 µL | APC | APC Hamster IgG | BioLegend | AB_492832 |
| CD90 | 0.6 µL | PerCP-Cy5.5 | PerCP-Cy5.5 Rat IgG 2b,k | BioLegend | AB_2571945 |
| Alkaline Phosphatase | 1 µL | APC | APC mouse IgG1 | R&D Systems | AB_2892551 |
| CD11b | 0.5 µL | FITC | FITC Rat IgG2b,k | BioLegend | AB_312789 |
| CD115 | 1 µL | APC | APC Rat IgG2a, K | BioLegend | AB_2085221 |
| CD68 | 1 µL | PerCP-Cy5.5 | PerCP-Cy5.5 Rat IgG 2b,k | BioLegend | AB_2260046 |
| RANKL | 1 µL | PE | PE Rat IgG2a,k | BioLegend | AB_2303587 |
| GP38 | 1 µL | PE | PE Hamster IgG | BioLegend | AB_2161929 |
| SPARC | 1 µL | APC | APC Rat IgG2b,k | R&D Systems | AB_2892552 |

## 2 Supplementary Figures


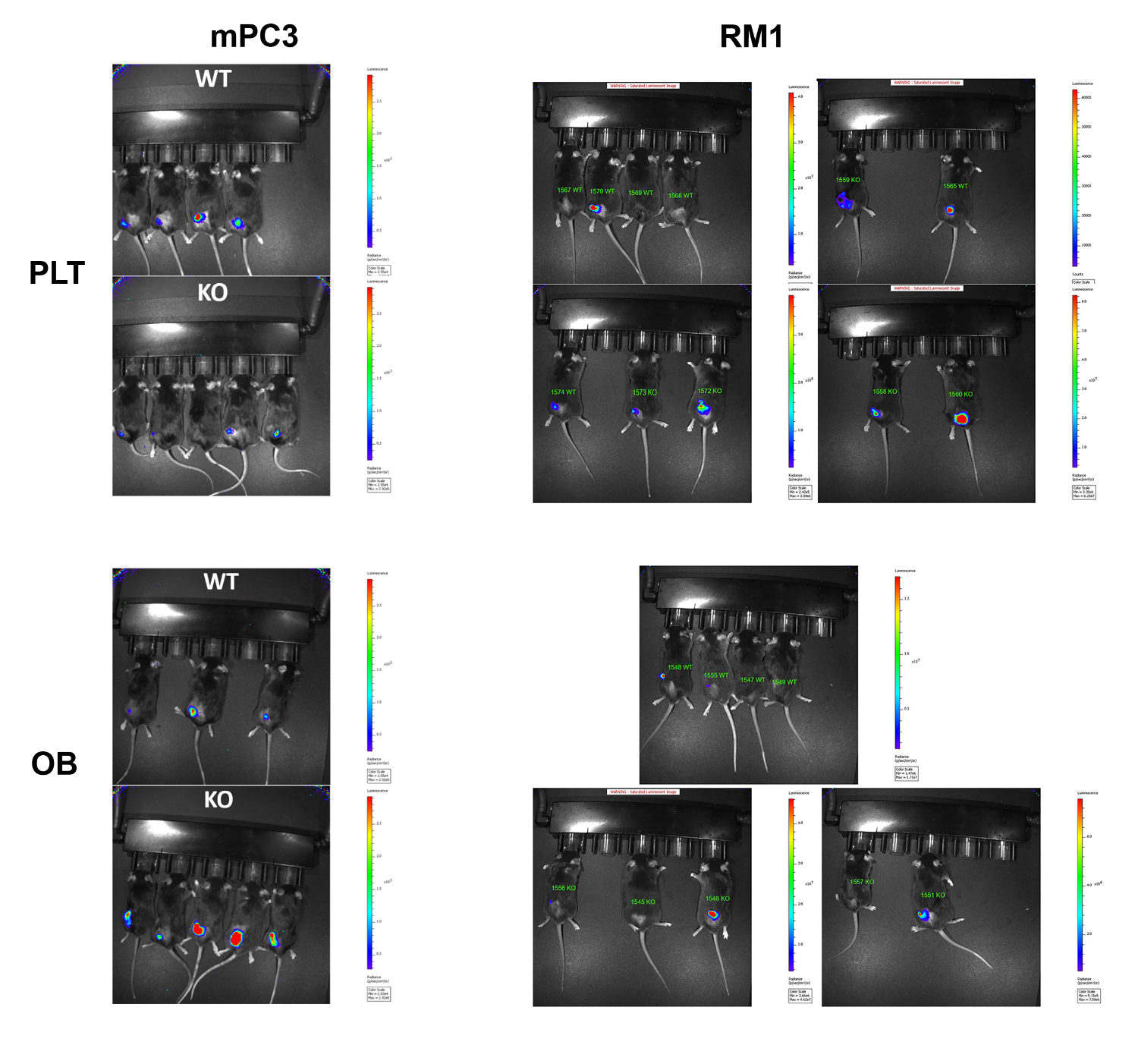


**Supplemental Figure 1. Radiance was not significantly altered after SCF Deletion.** mPC3 and RM1 cells were injected subcutaneously into PLT-WT, PLTΔSCF, OB-WT, or OBΔSCF mice. Tumors were allowed to grow for 12 days and imaged via IVIS on day 11 for average radiance


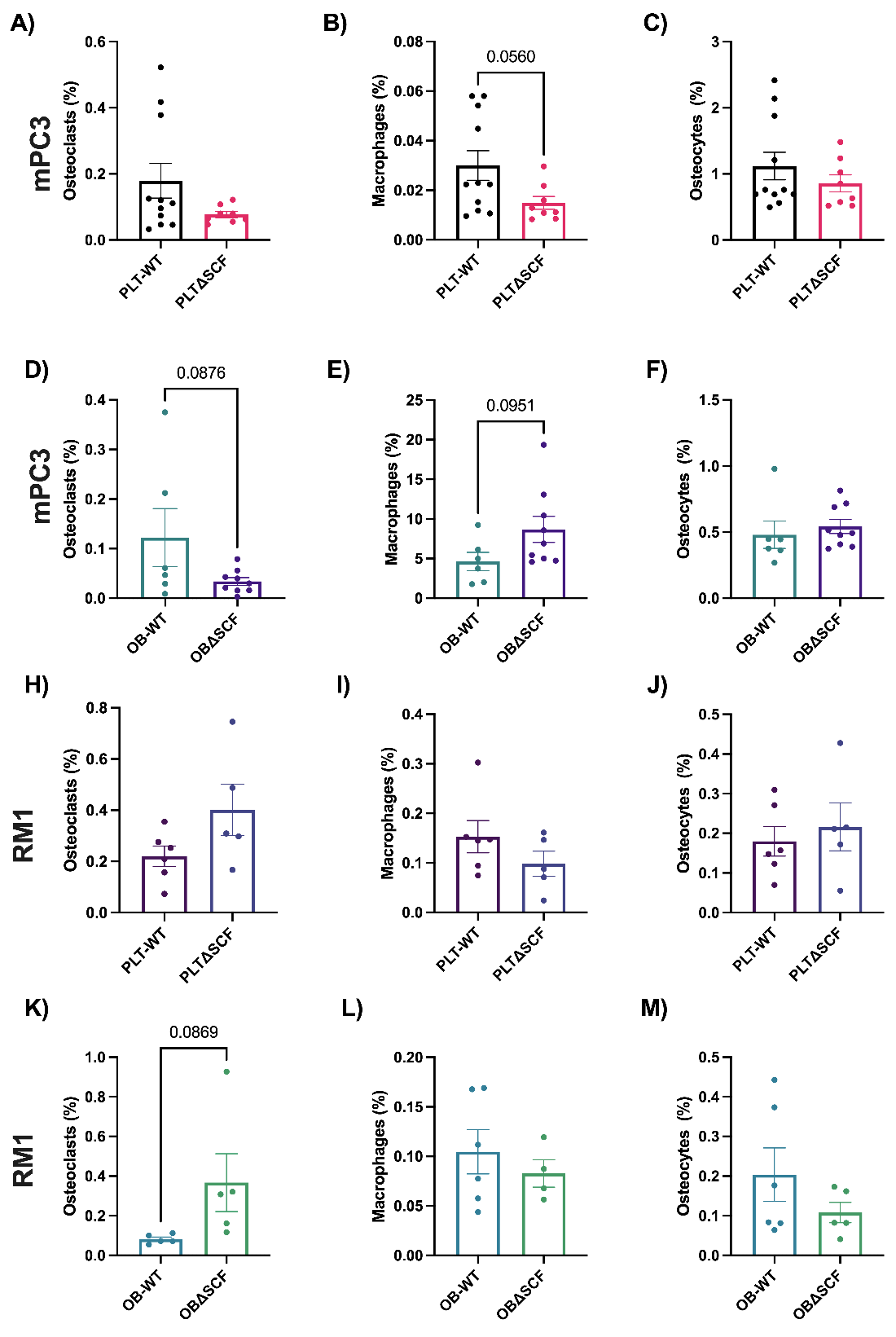


**Supplemental Figure 2. SCF does not affect osteoclast, macrophage, or osteocyte bone stromal cell populations.** Tibiae were isolated from PLT-WT, PLTΔSCF (A-C and H-J), OB-WT, or OBΔSCF (D-F and K-M) mice after tumor implantation with mPC3 (A-F) or RM1 (H-M) prostate cancer cells. Bone marrow was isolated and stained for osteoclasts (A, D, H, K), macrophages (B, E, I, L), or osteocytes (C, F, J, M). Flow cytometry was performed to calculate percent cell population. Represented as ±SEM (n=6-9).
